# A Perceived Zone of Certainty and Uncertainty: Propositions for Research Development

**DOI:** 10.3389/fpsyg.2021.666274

**Published:** 2021-09-08

**Authors:** Huy P. Phan, Bing H. Ngu

**Affiliations:** School of Education, University of New England, Armidale, NSW, Australia

**Keywords:** cognitive entrenchment, comfort zone, optimal best, cognitive certainty, flow, consonance of best practice, positive psychology, optimization

## Abstract

Possessing expert schemas is a positive feat that may yield different types of adaptive outcomes (e.g., informing procedural understanding that may result in a student skipping a few of the solution steps involved). Limited schemas, in contrast, may deter progress of a novice learner, limiting his/her capability to flourish. Taken as a whole, it may be concluded that expert schemas are more advantageous than novice schemas, differentiating learners in terms of expert and novice. Having said this, however, more recently, researchers have argued that possessing expert schemas could serve as deterrence. Recently, researchers have acknowledged a theoretical concept known as *cognitive entrenchment*, which is defined as a high level of stability in domain schemas. This description interestingly suggests that “entrenchment” or “situated fixation” of a course of action (e.g., a subject matter) could hinder the progress and learning experience of a person, namely—his/her inability and/or unwillingness to adapt to a new context, and/or his/her inflexibility and insistence to stay on course without any intent to change. One example of cognitive entrenchment is observed in professional football, wherein it has been argued that some football coaches are cognitively entrenched within their expert schemas, resulting in their demised game plans and strategic acumen. We advance the study of cognitive entrenchment by proposing an alternative viewpoint, which we term as the “perceived zone of certainty and uncertainty.” This proposition counters the perspective of cognitive entrenchment by arguing that it is cognitive appraisal, judgment, mental resolute, and determination of a person in *cognitive certainty* of his/her success or failure, or the *cognitive uncertainty* of success or failure, that would explain the notion of inflexibility and/or unwillingness to adapt, and/or insistence to stay on course without any attempt to deviate. Moreover, we rationalize that certainty of success or failure would closely associate with a feeling of comfort, whereas uncertainty would associate with his/her feeling discomfort. In this analysis, we strongly believe that willingness to change and adapt, reluctance and insistence to remain on course, and/or inclination to embrace flexibility may not necessarily relate to the concept of cognitive entrenchment; rather, inflexibility and/or reluctance to change for the purpose of adaptation has more to do with the desire of a person to seek a state of comfort. Finally, our conceptual analysis of cognitive entrenchment also considers an interesting theoretical concept, which we termed as “perceived optimal efficiency.” Perceived optimal efficiency, similar to *cognitive relevance theory*, is concerned with the relationship between minimum investment of time, effort, cognitive resources, etc., and an optimal best outcome. The issue for discussion, from our point of view, is related to the extent to which the certainty of success or failure would associate with perceived optimal efficiency.

## Introduction

Recently, an article has been published, titled “*Reconsidering the Trade-off Between Expertise and Flexibility: A Cognitive Entrenchment Perspective*” (Dane, 2010), which introduced a term known as “cognitive entrenchment” (Dane, 2011; Schmid, 2017; Engelberg, 2018). Cognitive entrenchment, in brief, is concerned with “a high level of stability in the domain schemas of an individual” (Dane, 2010, p. 583). This description interestingly emphasizes in-depth knowledge and understanding of a person in a subject matter and, more importantly, how this expertise could influence his/her willingness to change and/or ability to adapt. Consider, for example, a secondary school student who engages in “repeated practicing of mathematics problems” (e.g., spending 1 h after school each day to solve arithmetic problems with two unknowns, *x* and *y*). This cognitive behavior (i.e., practicing as many problems as possible) may eventually result in automaticity, enabling the student to successfully solve similar problems without any difficulty. *Automaticity*, reflecting rote learning, memorization, and excessive practicing, may be viewed as an indication of deep, meaningful understanding of a subject matter, making it somewhat difficult to discount. In this sense, being able to utilize a cognitive strategy without apprehension and/or any indication of uncertainty would, for some, connote evidence of expertise.

Automaticity, success, and ease may all combine to explain and account for cognitive entrenchment (e.g., the unwillingness of a person to change). This theoretical premise is logical as it suggests that a person may feel more inclined to stay on course when he/she is well-versed and knowledgeable about a subject matter. Largely, in terms of observation and explanation, a person may feel more at ease with the *status quo* than to change course, given the probability of his/her uncertainty of success, etc. On this basis, expertise instills confidence, mental resolute, and self-determination, convincing a person to maintain and sustain his/her course of action. Novice schemas, in contrast, could also act as sources of motivation, potentially compelling a person to consider changing his/her course of action in order to improve. From this brief account, we contend that inner satisfaction, gratification, and/or fulfillment of inner needs may explain “situated fixation” (i.e., for an expert) or change (i.e., for a novice) of a person to his/her own knowledge, understanding, skills, etc. This theoretical account is poignant as it suggests that there are potential reasons, which may account for cognitive thinking, action, and behavior of a person—for example, the willingness to change vs. the insistence to stay on course.

The focus of this article is for us to introduce and discuss an alternative perspective that may counter the propositions of cognitive entrenchment (Dane, 2010, 2011). We argue that there are a number of “positive” and/or valid reasons that may justify and/or explain the situated fixation of a person, and his/her reluctance to change in order to adapt to new contexts and situations. One particular reason or factor, in this case, relates to the quest of a person to *seek a state of comfort* (i.e., which is a positive facet) and, by the same token, to *avoid a state of discomfort* (i.e., which is a negative facet). For example, a university student may choose to remain on course with a specialization (e.g., a History specialization) despite the objection of his/her family, or the willingness of a musician to change genre may arise from his/her perception of comfort (e.g., the musician perceives it as being more comfortable) or a desire to seek comfort. With this premise in mind, we rationalize that there are a number of cognitive determinants that could associate with the seeking of comfort of a person—for example, *cognitive appraisal, judgment, decision-making*, and *determination* of “certainty” of a successful course of action. In contrast, likewise, we propose that a perceived sense of uncertainty would intimately link to a state of discomfort. In the latter section of this article, we discuss an important proposition that we term as the *principle of cognitive certainty and uncertainty*, which considers cognitive appraisal, judgment, and decision-making of a situated context in terms of comfort and discomfort.

## Introducing Cognitive Entrenchment: A Brief Overview

Relating to the study of *cognitive load imposition* (Sweller et al., 2011; Sweller, 2012) is an interesting inquiry known as *cognitive entrenchment* (Dane, 2011; Schmid, 2017; Engelberg, 2018), which is concerned with existing knowledge or schemas of a person. According to Dane (2010), cognitive entrenchment is defined as “a high level of stability in domain schemas of a person. The schema stability characterizing cognitive entrenchment may emerge, at least in part, from the frequency with which experts tend to draw on their domain schemas” (p. 583). The definition of cognitive entrenchment of Dane (2010) places emphasis on the importance of a high level of domain-specific knowledge and understanding of a person *via* means of personal experiences (Benner, 1984; Dreyfus and Dreyfus, 1986; Charness and Schultetus, 1999; Ericsson, 2006; Anders Ericsson et al., 2007). *Expertise*, or *expert schemas* (i.e., a high level of domain-specific knowledge of a person), is different from *novice schemas*, or inexperience and limited knowledge, skills, and/or understanding of and in a subject matter. A novice learner in this case, for example, would exhibit superficial understanding (e.g., being able to recall some facts without truly understanding) and have difficulties in comprehension and/or adaptation to a new context or a similar situation.

One major distinction between experts and novices is concerned with the *nature* of schema. The nature of schemas, in this case, delves into the complexity, or quantity and quality, of the schemas of a person (Fiske and Taylor, 1991; Rousseau, 2001). In this analysis, the totality of the “repertoire” of schemas for an expert exceeds that of a novice. For example, in terms of secondary mathematics learning, aside from deep, meaningful understanding of equation solving, an expert learner may have in-depth procedural knowledge of different pedagogical approaches (e.g., the expert learner may know the *balance method* and the *inverse method* of learning) (Ngu and Phan, 2016) as opposed to a novice learner, who may know only one pedagogical approach (e.g., the novice learner knows only the *balance method*). Interestingly, the work of Piaget (1963, 1990) illustrates a clear distinction between expert and novice schemas where, in this case, experts have more complex and interrelated schemas than novices. The “formation of schemas” (e.g., acquiring understanding of the concept of the black hole), according to Piaget (1963, 1990), consists of a personal experience known as “cognitive conflict,” or *cognitive disequilibrium*, which would require some form of resolution. In other words, when confronted with a new learning context that causes an unbalanced cognitive state, a person would make a concerted attempt to address this disconsonance; this resolution to transform an unbalanced cognitive state (e.g., not knowing how to solve 4*x* + 5 = −6) to a balanced cognitive state of thinking (e.g., knowing how to solve 4*x* + 5 = −6), importantly, reflects the cognitive growth of a person. In relation to academic learning, say, a student may use group discussions, debates, individual exploration, group work, teacher scaffolding, etc., to resolve his/her cognitive conflict (Phan and Ngu, 2019).

The theory of personal constructivism of Piaget (1963, 1990) contends that cognitive growth, which consists of the resolution of cognitive conflicts, reflects improved or acquired schemas in a subject matter (e.g., knowledge pertaining to Black Holes). In this analysis, cognitive growth is evident when one experiences and is able to resolve an unbalanced cognitive state of thinking. An internal cognitive state of stability or equilibrium over time, in contrast, would suggest limited, if any, cognitive growth in a subject matter. For example, in secondary mathematics learning, a student is well-versed in problem-solving of one unknown, *x*, but nothing else. As a person grows older, according to Piaget (1963, 1990), his/her schemas would become enriched and more intricate both in terms of quantity and quality. Over the past number of years, our research in Mathematics Education has shown that many secondary school students come to acquire relevant schemas of different pedagogical strategies (e.g., balance vs. inverse) that could, in effect, facilitate their understanding (Ngu and Phan, 2016).

There has been extensive research development into the contrasting nature of expertise and novice schemas. One notable inquiry that has been studied in detail, for example, relates to chess playing and how expert players differ from novice players (Gobet, 2006; Bilalic et al., 2008; Nokes et al., 2010; Lane and Chang, 2018). Expert players, in this case, are able to recognize and identify familiar patterns in chess positions, and they have knowledge and understanding of larger patterns. Another line of inquiry into the nature of expertise, which is closely aligned with our existing research development, is that of cognitive load imposition or cognitive load burden (Sweller et al., 2011; Sweller, 2012). Cognitive load imposition, in brief, is concerned with the imposition in cognitive processing of information that may arise from a complex subject matter and/or from an ineffective instructional design. In terms of academic learning, an ineffective instructional design would impose a high level of cognitive load, limiting the comprehension and understanding of a student of the involved subject matter (Ngu et al., 2016, 2018a,b). Cognitive load imposition is negative and in this sense, weakens the comprehension, understanding, and performance outcome of the student. Unlike that of a novice, an expert has relevant expertise (e.g., expert schemas), which would help to minimize cognitive load imposition.

Clearly then, there are benefits for having expert knowledge and skills in a domain of functioning. Experts with in-depth knowledge, skills, and understanding make effective decisions, exhibit superior recall of information, perform well, academically and/or non-academically, and are able to adapt and engage in problem-solving, which may transfer to different contexts (Chi et al., 1988; Hoffman, 1992; Ericsson et al., 2018). For example, in terms of academic learning of mathematics, a secondary school student who has expert knowledge and in-depth understanding of linear equations could potentially skip a particular step involved. This skipping of step or steps is of considerable interest, especially in terms of efficiency (e.g., cost effectiveness), which may involve the reduction and/or minimization in cognitive load imposition. In a similar vein, an expert learner is more inclined to seek mastery and deep, meaningful learning for the purpose of personal growth and/or skill improvement. A novice learner, in contrast, would have to spend more time, effort, etc., in order to comprehend and, hopefully, to understand the problem at hand. Furthermore, a novice learner is also likely to exhibit disorganization and/or unstructured learning habits, giving rise to under-achievements and negative learning experiences. By all accounts, in terms of comparison, an expert is well-placed to experience a high level of motivation and to achieve personal improvement, academic growth, etc. A novice with limited schemas, in contrast, is likely to struggle in terms of adaptation to a new learning context or situation. Interestingly, some scholars have contended that expert schemas could serve as deterrence, limiting a person from adapting and progressing further in his/her cognitive development. This acknowledgment is contentious as it suggests the plausibility that expertise in itself could detrimentally influence the learning processes. One potential deterrence forming the premise of this article relates to a theoretical concept and/or inquiry known as “cognitive entrenchment” (Dane, 2010, 2011). The term “entrenchment,” in this analysis, emphasizes the situated fixation of the mindset of a person to a particular context or course of action (e.g., insistence and continual usage of a student of a particular cognitive strategy in his/her learning). Moreover, the situated mindset of a person is contextualized within the specificity of time, cognitive task, personal circumstance, event, etc.—for example, insistence of a student (i.e., his/her situated mindset) to use a particular pedagogical strategy in order to solve a particular Algebra problem (i.e., the specificity of a cognitive task), or a favorable view of a senior citizen (i.e., his/her situated mindset) of multiculturalism, which, in this case, is shaped and guided by his/her personal experiences (i.e., the specificity of personal experience).

### The Nature of Cognitive Entrenchment

Situated fixation may limit the flexibility of a person to progress and grow cognitively and/or non-cognitively (e.g., Lewandowsky and Kirsner, 2000; Chi, 2006; Lewandowsky et al., 2007). The theoretical account of cognitive entrenchment of Dane (2010) connotes the tenet that having expert knowledge could, in fact, deter a person from engaging in creativity and/or innovation. Specifically, according to Dane (2010, p. 583), experts exhibit a restricted ability to accommodate new rules and principles (Frensch and Sternberg, 1989; Marchant et al., 1991) and that, likewise, they have difficulties in understanding how novices approach their problem-solving of problems (Camerer et al., 1989; Hinds, 1999; Thaler, 2000; Birch and Bloom, 2007). Moreover, in accordance with the perspective of cognitive entrenchment, expert schemas may confine the flexibility and willingness of a person to make proactive changes. In place, likewise, according to Dane (2010), cognitively fixating on the expertise one has, may instill a sense of inflexibility, which may consist of the following: (i) the *unwillingness* of a person to change his/her course of action or cognitive thinking, (ii) the *perceived difficulty* of a person to adapt, make changes, and/or resolve a new context or situation, and (iii) the *reluctance* of a person to accept change, criticism, and/or advice.

Upon inspection, there is credence to acknowledge and accept the “negativity” of cognitive entrenchment, which may apply to different contexts. One non-academic example of cognitive entrenchment recently discussed relates to professional football coaching. Those who follow European football would know the name José Mourinho, who is considered to be one of the most revered and decorated coaches of all time (source: https://sportsshow.net/most-successful-football-managers/). Many observers have noted that despite his stellar track record (e.g., winning 25 titles, including two prestigious UEFA Champions League titles), José Mourinho has somewhat declined to the point where some journalists and pundits have referred to him as a man of yesterday (source: https://www.the42.ie/is-jose-mourinho-now-yesterdays-man-5140367-Jul2020/). In a recent article published online, for example, Tannoury (2020) wrote the following: “On the pitch, the tactics employed by Mourinho—irritatingly defensive setups and opportunistic play in attack, with long passes launched for the wingers or the lone striker—have been left behind by rivals such as Jurgen Klopp at Liverpool and Pep Guardiola of Manchester City. Now, a younger generation of football managers, including 33-year-old Coach Julian Nagelsmann of RB Leipzig, are introducing new concepts that are evolving the game. Mourinho, so far, has not adapted” (source: https://www.thenational.ae/sport/football/twenty-years-as-a-manager-for-jose-mourinho-this-season-could-be-his-most-important-yet-1.1086662). This collective insight, interestingly, reflects a potential example of cognitive entrenchment for José Mourinho, detailing his inability, inflexibility, and/or unwillingness to adapt to the “modern game” of football. An article of Grech (2020) titled “*Cognitive Entrenchment and the Curious Case of José Mourinho*” (source: https://footyanalyst.com/cognitive-entrenchment-and-the-curious-case-of-jose-mourinho/), likewise, has provided an in-depth analysis of demise of José Mourinho and in particular, his personal experience of cognitive entrenchment. Concurring with assessment of Liliane Tannoury, Paul Grech argues that the modern game in football has evolved and the training methodology of José Mourinho (i.e., the methodology of what is known as “tactical periodization”), which served so well has become obsolete.

Non-academically, as the case of José Mourinho has shown, expertise in a particular domain of functioning (e.g., a particular training methodology in football) may limit the inclination and/or willingness of a person to engage in inventive, innovative, and/or creative acts. Cognitive entrenchment, according to Dane (2010, 2011), may instill a fixed mindset, which would deter a person from adapting to a new context. Academically, for example, cognitive entrenchment may limit understanding and/or acceptance of a student of different pedagogical approaches (e.g., the *inverse method* and the *balance method*) (Ngu and Phan, 2016) and, hence, his/her usage of an effective pedagogical approach, which could facilitate effective learning experiences. By the same token, of course, situated fixation to a course of action (e.g., a student fixating on her knowledge) could deter a student from choosing a new course of action for learning. Despite this consideration, we rationalize that cognitive entrenchment could have a number of valid and positive reasons (e.g., the positive effect of cognitive entrenchment), refuting original propositions of Dane (2010, 2011) that entrenchment of domain-specific expert knowledge is negative and detrimental.

Valid and positive reasons for the enactment of cognitive entrenchment in both educational and non-educational contexts are plausible. This consideration is poignant as it posits the possibility that one may purposively “fixate” to a course of action for meaningful reasons. One interesting reason, for example, may relate to the internal desire of a person to experience a “state of comfort” rather than discomfort. To date, to our knowledge, very little has been inquired into the extent to which seeking of comfort of a person could account for his/her cognitive entrenchment. As a possibility, unwillingness to change from the *status quo* (e.g., insistence of a university student to remain with a particular degree program despite her continuing failures) may arise from a belief of a person that such “deviation” would cause chaos and result in a perceived state of uncertainty. Moreover, cognitive entrenchment may instill and/or strengthen the confidence and state of mental resolute of a person, assisting to account for a perception of comfort.

### A State of Comfort: An Introduction

The preceding sections have provided a brief overview of cognitive entrenchment (Dane, 2010; Schmid, 2017; Engelberg, 2018) and its proposed state of negativity. Having said this, however, a question that we could ask is whether and/or to what extent there is justification to portray cognitive entrenchment as being negative? We contend, as briefly described, that the unwillingness of a person to change (i.e., the action of cognitive entrenchment of the person) may arise from and/or associate with meaningful reasons and purposes. We choose one interesting aspect for discussion, which has been referred to in the literature as the *perception of comfort* of a person (White, 2009; Liepold et al., 2013). What is a perception of comfort? And how does this potentially account for a cognitive entrenchment? Perception of comfort is intricately linked to what is known as a “zone of comfort” or a “comfort zone,” which is defined as “a behavioral state within which a person operates in an anxiety-neutral condition, using a limited set of behaviors to deliver a steady level of performance, usually without a sense of risk” (White, 2009, p. 2). This definition of a comfort emphasizes the emotions of a person, preferably positive (e.g., a state of happiness). Moreover, definition of White (2009) contends that the level of performance of a person would remain constant in the absence of a change in anxiety and/or any other negative emotion. By the same token, a change in the level of anxiety and/or any other negative emotion would influence the performance of a person—either downwards or upwards. Existing research has shown, for example, that anxiety is negatively associated with academic performance and achievement of other adaptive outcomes (Pajares and Kranzler, 1995; El-Anzi, 2005; Segool et al., 2013; Onyekuru and Ibegbunam, 2014). Positive emotions (e.g., a state of joy), in contrast, would give rise to a perception of comfort, resulting in the improved performance of a person. In a similar vein, there is evidence attesting to the association between positive emotions (e.g., happiness) and an improvement in academic performance (Spice, 2011; Tabbodi et al., 2015; Phan and Ngu, 2020).

We contend that a state of zone or being “situated” within a comfort zone, as White (2009) defines, may coincide with the *paradigm of positive psychology* (Seligman, 1999; Seligman and Csíkszentmihályi, 2000) and, relatedly, the psychological concept of “flow” (Csíkszentmihályi, 1997, 2014a,b). A state of comfort, we rationalize, is positive and may entail and/or emphasize the importance of personal growth, positive emotions (e.g., a state of contentment), and enriched experiences. Comfort, likewise, may also espouse a “flow state” or a “flow zone,” motivating a person to flourish and to achieve individual growth in a subject matter. This equivalency (i.e., comfort zone ↔ positive flow state), taking into account the theory of flow of Csíkszentmihályi (2014a,b) considers an interesting “growth ratio”—namely, skill level > challenge level (i.e., the skill level of a person or knowledge exceeds that of the level of challenge of a course of action).

A state of discomfort, we rationalize, is negative and may entail and/or emphasize the importance of negative emotions (e.g., a state of anxiety or apprehension), stagnated progress, and limited, if any, growth. Taking into account the theory of flow of Csíkszentmihályi (2014a,b), we contend that a state of discomfort may also espouse an interesting “stagnated growth ratio”—namely: challenge level > skill level (i.e., the level of challenge of a course of action exceeds that of the skill level or knowledge of a person). Moreover, of course, the equivalency of discomfort or a discomfort zone and a negative flow state (i.e., discomfort zone ↔ negative flow state) is detrimental, reflecting suboptimal experiences, academically and/or non-academically, and feelings of pessimism and helplessness.

It is natural for us to want to seek a state of comfort and, by the same token, to avoid a state of discomfort. In academic and/or in school contexts, for example, we contend that a comfort zone is intricately linked to a perceived positive school climate, and/or a classroom climate, espousing the perceptions, feelings, and experiences of emotional support, social safety, academic scaffolding, etc. (Roorda et al., 2011). One interesting aspect that may instill and facilitate a positive perception of comfort relates to a well-developed teacher-student social relationship, commonly known as TSR (Bergeron et al., 2011; Allen et al., 2013; Gallagher, 2013). A positive TSR, in this instance, would motivate children to feel at ease and autonomous in the teaching and learning processes. We rationalize that a negative TSR (e.g., the perception of a child that his/her teacher is not supporting him/her) would, in contrast, create and/or account for a state of discomfort, instilling feelings of pessimism, helplessness, negativity, etc., which could result in the unwillingness of a child to engage in academic learning and/or extracurricular activities. In essence, we expect that children would seek out and prefer to experience comfort, the feeling of ease, motivation, etc., and, by the same token, to avoid discomfort, angst, uneasiness, unpleasant occurrences, etc. As such, between the two contrasting zones (i.e., the zone of comfort vs. the zone of discomfort), we are more inclined to seek and orientate toward the comfort zone and to avoid the discomfort zone as the former is perceived as being more pleasant, positive, harmonious, etc.

## Potential Association Between Cognitive Entrenchment and Comfort Zone

One underlying premise of our examination and focus of inquiry entails the plausibility that cognitive entrenchment, or the situated fixation of a person to a well-versed course of action, could intimately relate to a state of comfort. For example, in-depth knowledge of a subject matter may instill a perception of stability, confidence, and optimism in a person, all of which are positive characteristics of comfort. Deviating from a well-versed course of action (e.g., a student may choose a different specialization), in contrast, could give rise to a perception of uncertainty, unsureness, and pessimism, coinciding with a sense of discomfort. The case of José Mourinho (source: https://footyanalyst.com/cognitive-entrenchment-and-the-curious-case-of-jose-mourinho/), as we previously discussed, is a potential example, which could substantiate our rationalization. In this analysis, the inflexibility of José Mourinho (i.e., the use of tactical periodization, which focuses on a defensive mindset) and his unwillingness to change (e.g., consideration to focus on setting up an attacking formation) may relate to a personal need for comfort—that he is more “comfortable” to use defensive techniques, which have brought him so many successes in the past.

From the preceding introduction, we argue that there is validity and justification for the enactment and testament of cognitive entrenchment (e.g., the reluctance of a university student to change specialization). In this analysis, we do not view the notion of cognitive entrenchment, or the situated fixation of a person to a course of action, as being negative and/or detrimental—for example, a person's unwillingness and/or reluctance of a person to change a course of action, and/or his/her indication of difficulty to adapt to a new context or situation. Our proposition, in this case, considers the potential positivity or positive “reasons” for the seeking of a person to remain fixated to a course of action. One sound and logical reason, as we described, is associated with the desire of a person to seek a state of comfort and, by the same token, to avoid a state of discomfort. In other words, situated fixation to a well-versed course of action (e.g., insistence of a secondary school student to use a particular pedagogical strategy) may continue to bring success, resulting in a perceived state of comfort (e.g., the experience of contentment). Deviating from a well-versed course of action, in this analysis, could instill unsureness and uncertainty (e.g., will I succeed if I use another pedagogical strategy that I am not well-versed in?), giving rise to feelings of angst, pessimism, and helplessness. In this sense, striving to achieve a perceived state of comfort would serve as reinforcement, whereas avoidance of discomfort would serve as deterrence to remain on course without any change (e.g., the reluctance of a person to use a new strategy).

## An Alternative Proposition: A Case For a “Perceived Zone of Cognitive Certainty” and Avoidance of “Uncertainty”

**Figure 1:**
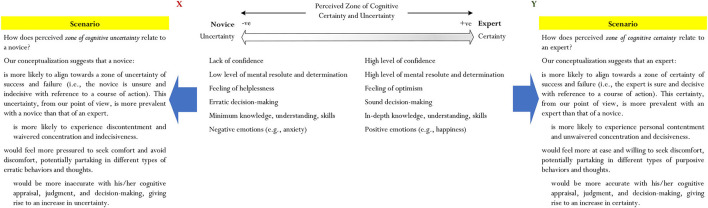
A perceived zone of cognitive certainty and uncertainty.

Our conceptualization for development, taking into account the theoretical concept of comfort (and discomfort) (White, 2009; Liepold et al., 2013) considers a theoretical term, which we coin as a “perceived zone of cognitive certainty and/or uncertainty.” As shown in Figure 1, we conceptualize and theorize that a *perceived zone of cognitive certainty* would align with a state of comfort, whereas a *perceived zone of cognitive uncertainty* would align with a state of discomfort. Foremost from this consideration is the importance of the assessment, judgment, and rationalization of a person, which could warrant and provide justification for the position of cognitive entrenchment (e.g., the choice of a person to remain on course with a particular action). On this basis, our consideration entails the question of whether one is *certain* (e.g., “I am certain that I will be successful….”), or *uncertain*, that a continuing course of action would bring success (e.g., with the case of José Mourinho, what is the certainty that his continual usage of periodization would result in success?).

We prefer the terms “certainty” (e.g., “I am certain that….”) and “uncertainty” (e.g., “I am uncertain that….”) as these nomenclature, we believe, would reflect the cognitive maturity of a person, detailing his/her understanding, skills, and experiences of judgment, assessment, and decision-making. For example, there are two possibilities: (i) the ability of a person to make sound and accurate judgment and assess whether a current course of action, drawn from existing schemas, would result in successful outcome(s), and (ii) the mental fortitude, state of confidence, and rationalization of a person to weigh his/her decision regarding the course of action.

From our point of view, the cognitive maturity of a person may consist of his/her individual assessment, judgment, and decision-making prior and during the course of an action. Our conceptualization, in particular, considers the extent to which assessment and judgment of a state of certainty or uncertainty (e.g., an undergraduate student choosing to major in History) could, in fact, account for and/or influence the situated fixation of a person to a course of action. We interestingly make attempts to unify and relate the following: (i) the zone of cognitive certainty with a perceived sense of comfort (i.e., cognitive certainty ↔ perceived sense of comfort), and (ii) the zone of cognitive uncertainty with a perceived sense of discomfort (i.e., cognitive uncertainty ↔ perceived sense of discomfort).

Cognitive certainty and cognitive uncertainty, we contend, are closely aligned with a number of theories—for example, the *theory of probability* (Athreya, 2015; Debnath and Basu, 2015; Seidenfeld, 2015), the *theory of risk-taking*, and the *theory of decision-making* (Igra and Irwin, 1996; Kusev et al., 2017; Zinn, 2019). In this analysis, deciding a state of certainty or uncertainty reflects, in our view, the statistical premise of probability—that is, the certainty of success vs. the certainty of failure is a 50–50 probability chance (i.e., 50% of certainty vs. 50% of uncertainty). This statistical probability is unambiguous and consists of two distinct possibilities: “I am certain that I will be successful” (50% chance) vs. “I am certain that I will fail” (50% chance) or, alternatively, “I am uncertain that I will be successful” (50% chance) vs. “I am uncertain that I will fail” (50% chance). Aside from statistical probability, it is also an important feat for a person to finalize and decide on a definitive course of action. In this analysis, the amount of time or effort that a person spends in order to achieve an optimal outcome is largely influenced by his/her perceived value of the desired outcome (e.g., Algebra is perceived as being important for entry into university), the influence of external factors (e.g., the explanation of a teacher and insight into the relevance and importance of investment of time), etc. For example, emphasis and valuing of a senior citizen of optimal health well-being may compel him/her to strongly commit, such as excessive investment and expenditure of effort and perseverance. This determination, reflecting the resolute decision-making of a person emphasizes some form of mental fortitude, personal resolve, and risk-taking. In this sense, does a person have the resolute mindset (e.g., a sense of self-determination) to take risk and, hence, make a logical decision, which could account for his/her course of action?

From the preceding sections, the testament of cognitive certainty of success, or failure of a person, may intimately relate to his/her assessment and judgment, and, more importantly, reflect the process of decision-making. The resolute or irresolute attitude of a person, for that matter, entails some form of risk-taking, which, in this case, consists of a 50–50 probability chance (i.e., the probability or chance of certainty of success or failure is 50–50). Risk-taking, however, is more than just a “present-moment” sense of determination and decision-making. Rather, despite its nomenclature, risk-taking entails the personal characteristic “considered weighing” of a person, which we define it as being his/her *cognitive appraisal* of a context at hand and weighing it up in terms of positivity vs. negativity—that is, whether the positivity would outweigh the negativity, or *vice versa*. The considered weighing of a person, in this case, emphasizes his/her analysis of two interrelated entities: cost vs. benefit—for example, is it worth the risk to change course and adopt a new approach to learning? In essence, considered weighing into the complexity of cognitive certainty and cognitive uncertainty (e.g., certainty over that of uncertainty) is thoughtful, timely, and purposive, serving as evidence of the state of cognitive growth and life wisdom of a person.

### The Nature of Certainty and Uncertainty of Success

Cognitive certainty, as we proposed, refers to the assessment, judgment, and rationalization of a person of a context or situation at hand, and whether his/her continual course of action would yield a positive outcome (i.e., certainty) or a negative outcome (i.e., uncertainty). This proposition, importantly, emphasizes the cognitive appraisal, mental resolute, and self-determination of a person to take risks and make sound, logical decisions. Risk-taking is an anticipatory feat that could have profound contrasting influences on a person—for example, in terms of positivity, the risk-taking of a university student may facilitate and/or improve his/her mastery of a subject matter, resulting in a state of cognitive certainty, confidence, resolute, and optimism. By the same token, of course, risk-taking may also produce negative returns (e.g., risk-taking of a secondary school student to choose an assessment topic that is somewhat difficult, resulting in a modest grade), giving rise to a state of cognitive uncertainty, pessimism, and low confidence.

Details pertaining to the nature of certainty and uncertainty are shown in Figure 1, depicting cognitive certainty and uncertainty on opposite ends of a continuous spectrum. This proposition, interestingly, does not differentiate cognitive certainty and uncertainty as two distinct entities with a reference point of neutrality in between (i.e., a reference point that may be denoted as “0”). From our point of view, we rationalize that a person could, in fact, transpose between a state of certainty and a state of uncertainty, depending on his/her cognitive appraisal, judgment, and decision-making. As an example, the cognitive appraisal, weighing, and self-determination of a person may convince him/her of a particular course of action, which could connote two contrasting possibilities:

i. There is cognitive certainty that a positive outcome (e.g., employment prospect for a university student) would be achieved with the continuation of a course of action (i.e., continuation of a university student to undertake Psychology).
ii. There is cognitive uncertainty that a positive outcome (e.g., success of winning of a football team) would be achieved with the continuation or a change in the course of action of a person (e.g., the decision of a football coach to change his/her training methodology).

Our conceptualization contends the possibility that a person could, in fact, transpose or “fluctuate” between a state of cognitive certainty and a state of cognitive uncertainty. A context at hand (e.g., the context of a secondary school student having to learn how to solve linear equations) and the subsequent approach of a person to this context, based on his/her existing schemas, may result in two contrasting positionings—that is: a negative position (i.e., denoted as –ve), which depicts the position of cognitive uncertainty of a novice vs. a positive position (i.e., denoted as +ve), which depicts the position of cognitive certainty of an expert. We posit that it is not a clear-cut 50–50% chance or probability of risk-taking and self-determination between cognitive certainty and cognitive uncertainty but, rather, as shown, an overlap between the two states. Consider the context of academia in which a university student uses his/her acquired knowledge, life wisdom, and the experiences of other students to change from a state of cognitive uncertainty to that of cognitive certainty. Over time, of course, his/her resolute, decisiveness, and cognitive certainty that Psychology, as a major, would bring positive returns (e.g., excellent job prospect) could change to one irresolute, indecisiveness, and cognitive uncertainty. In another context, likewise, a Year-9 student may indicate a state of cognitive uncertainty when learning a topic in Algebra where, over time, with continuing practice and improvement in mastery of using different pedagogical approaches, he/she is able to change his/her mental resolute, conviction, and belief in one of the cognitive certainties. Thus, from our rationalization, we stipulate the following possibilities:

i. ***A perceived zone of cognitive certainty***. Cognitive certainty, or a perceived zone of cognitive certainty, is positive and may, in fact, equate to that of a perceived state of comfort. We define *cognitive certainty* as an “envisaged state of decisiveness of a person, reflecting his/her confidence, mental resolute, and self-determination that a chosen course of action would yield either success (e.g., I am certain that a change in the course of action will bring success) or failure (e.g., I am certain that continuation with this course of action would yield failure).” Moreover, we speculate that expertise, unlike novice knowledge, could instill confidence, an appropriate level of motivation, and mental resolute, which, in effect, would determine the cognitive certainty of a person.
  Expertise is advantageous as this would assist a person to remain unchanged during the course of an action, which, in turn, could result in his/her achievement of success or his/her recognition of potential failure. On this basis, we postulate that expert learners with their in-depth knowledge and understanding would more likely associate with a state of cognitive certainty than that of cognitive uncertainty. Importantly, from our point of view, a state of cognitive certainty of success (e.g., I am certain that I will succeed with the continuation of this course of action) or a state of cognitive certainty of failure (e.g., I am certain that I will fail and not succeed if I proceed with a change in direction) would indicate some form of “finalization,” giving rise to perceived feeling of comfort of a person. In other words, from our proposition, a state of cognitive certainty is more “definitive” and “conclusive,” whereas a state of cognitive uncertainty is indefinite and inconclusive, giving rise to a feeling and experience of discomfort, angst, pessimism, etc.
ii. ***A perceived zone of cognitive uncertainty***. Cognitive uncertainty, or a perceived zone of cognitive uncertainty, is negative and may equate to a perceived state of discomfort. We define *cognitive uncertainty* as “an envisaged cognitive state of indecisiveness of a person, reflecting his/her lack of confidence, hesitation, ambivalence, and questionable thoughts that a course of action would yield either success (e.g., I am certain that a change in the course of action will bring success) or failure (e.g., I am certain that continuation with this course of action would yield failure).” Moreover, we speculate that cognitive uncertainty reflects the weak mindset of a person, which may espouse a low level of self-belief, mental resolute, and self-determination in terms of decision-making. We speculate that, unlike expert learners, a novice learner is less certain, less resolute, and less confident in his/her cognitive appraisal of a course of action.
  Moreover, unlike that of cognitive certainty, we propose that cognitive uncertainty may closely align with the perceived feeling of discomfort of a person. Importantly, of course, the limited knowledge and understanding of a subject matter could cause a perceived sense of indecisiveness, reflecting a state of hesitation, ambivalence, and questionable thoughts about the extent to which a person could succeed. In this analysis, from our point of view, a person is more likely to perceive a state of discomfort when he/she adheres and/or expresses a state of cognitive uncertainty. In the context of schooling, a secondary school student who has limited content and procedural knowledge of Algebra, for example, is more likely to express a state of cognitive uncertainty (e.g., the student is uncertain of whether she will succeed) and, correspondingly, a feeling of discomfort (e.g., the feeling of angst).

### Summary

From our examination, cognitive certainty is positive and may equate to the feeling of comfort, whereas cognitive uncertainty is negative and may equate to the feeling of discomfort. We propose that, progressively, with changing knowledge, skills, and experiences, decisiveness (or indecisiveness), mental resolute, and determination may change, which could result in a shift from a state of cognitive certainty to that of cognitive uncertainty, or from a state of cognitive uncertainty to that of cognitive certainty. A person, likewise, may alter and shift his/her feeling of comfort to that of discomfort, or *vice versa*, correspondingly reflecting a state of cognitive certainty or a state of cognitive uncertainty. What is of interest, however, is the possibility that cognitive certainty and cognitive uncertainty may situate and coexist within a dynamic spectrum.

Natural tendency would indicate that, perhaps, we all desire the personal feeling and experience of comfort in life. Comfort, unlike that of discomfort, is positive (e.g., comfort may give rise to a state of contentment and happiness) and produces and/or causes an improvement in different types of adaptive outcomes (e.g., academic performance). Cognitive certainty of success, or cognitive certainty of failure, is conclusive and more definitive, which may account for a feeling of comfort. Cognitive uncertainty of success, or cognitive uncertainty of failure, in contrast, is inconclusive and indefinite, giving rise to a feeling of discomfort, doubt, apprehension, etc. Importantly, from our point of view, both cognitive certainty and cognitive uncertainty may, in fact, associate with the theoretical concept of entrenchment (Dane, 2010, 2011). The inclination of a student to cognitively fixate on a subject content and/or course of action (e.g., a student fixates on a specific pedagogical strategy that he/she is well-versed in), for example, may help improve his/her learning experiences of a topical theme, resulting in a heightened state of mental resolute, decisiveness, and self-determination (i.e., a state of certainty).

**Figure 2:**
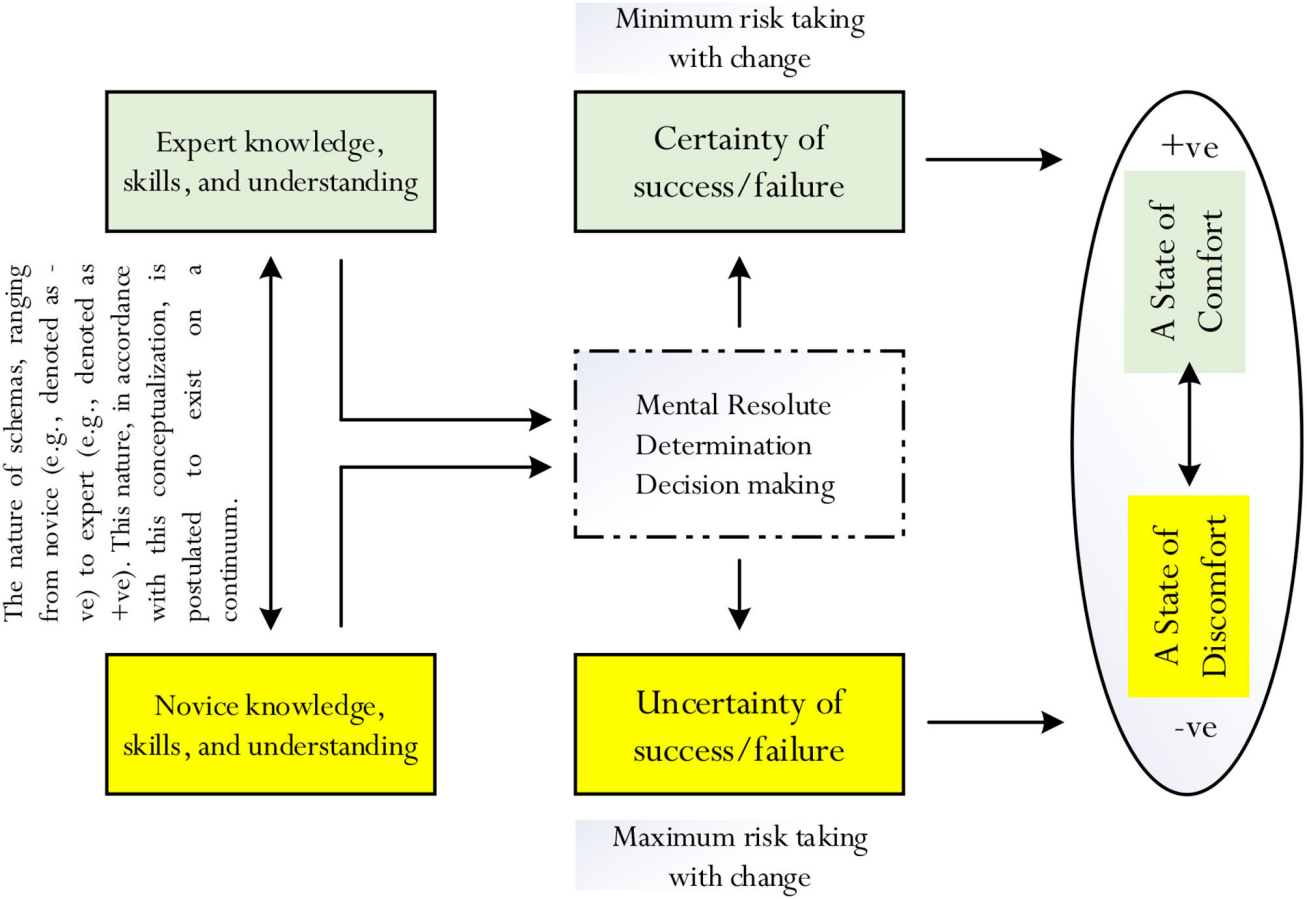
Summary of proposition. This is a summarized depiction of our proposed concept of the zone of cognitive certainty and the zone of cognitive uncertainty. The distinction between expert and novice knowledge is postulated to act as an important driver, which would direct and govern the mental resolute, determination, and decision-making of a person. For example, as identified by the coloring in this figure (i.e., yellow vs. green), a person with expert knowledge, skills, and understanding is more likely to be resolute, determined, and decisive in being certain of his/her success or failure, resulting in the feeling and experience of comfort (i.e., the green pathway). In contrast, likewise, a person with limited knowledge and understanding is less likely to be resolute, determined, and/or decisive in terms of certainty, resulting in the feeling and experience of discomfort (i.e., the yellow pathway).

Our proposition, indeed, offers an alternative insight into the potential positivity of the cognitive entrenchment of a person (Dane, 2010, 2011). Differing from the theoretical account of Dane (2010), we propose that fixation of a person to a subject matter and/or course of action (e.g., the unwillingness of a student to change his/her major in Chemistry) could intimately associate with a state of cognitive certainty and, similarly, account for his/her desire to seek a state of comfort. The conviction of José Mourinho of his training methodology approach and the perceived cognitive certainty that this would bring continuing success (and, hence, his feeling and experience of comfort) could, in fact, explain why he chooses to remain “unchanged.” Our conceptualization, as shown in Figure 2, considers the following: a *difference* in knowledge, skills, and understanding (i.e., expert vs. novice) could act as a central driver, which in turn would help govern the mental resolute, self-determination, and state of decisiveness of a person. Moreover, in accordance with our proposition, there are two contrasting zones that a person may purposively choose: the zone of cognitive certainty, potentially giving rise to a state of comfort, which is positive vs. the zone of cognitive uncertainty, potentially giving rise to a state of discomfort, which, of course, is negative.

## The Concept of Perceived Optimal Efficiency

Our research inquiries into the topic of *optimal best practice* (e.g., Phan et al., 2016, 2017, 2019a,b) have led to our recent development of a theoretical concept known as “perceived optimal efficiency” (Phan and Ngu, 2021c), which emphasizes the importance of an analysis of cost (e.g., time, effort, resources, etc.) vs. benefit. The nature of perceived optimal efficiency, we contend, is similar to the study of *relevance theory* (Sperber and Wilson, 1986, 1995), which focuses on two major principles: (i) the *cognitive principle of relevance* and (ii) the *communicative principle of relevance*. The cognitive principle of relevance, interestingly, indicates that internal cognitive processes of a person (e.g., his/her memory span) are guided by his/her consideration of efficiency (Wilson and Sperber, 2004). For example, within the context of academic learning, a university student is more likely to attempt to allocate cognitive resources (e.g., memory, attention) that would yield maximum cognitive effect for the least processing investment. It does not make logical sense for the student to allocate cognitive resources that would, in this case, result in minimum cognitive effect for the most amount of processing investment. This brief description of relevance theory, indeed, places emphasis on two interrelated entities: investment (e.g., allocation of cognitive resources) and outcome (e.g., maximization in cognitive effect).

Perceived optimal efficiency (Phan and Ngu, 2021c), similar to that of relevance theory (Sperber and Wilson, 1986, 1995), has been conceptualized to help explain the experience of optimal best of a person in a subject matter (Fraillon, 2004; Liem et al., 2012; Phan et al., 2016), which emphasizes his or her maximization in functioning (e.g., physical functioning)—for example, a professional football player may indicate that his optimal best in scoring for the 2021/2022 season is 85 goals (Phan et al., 2020). In a similar vein, non-academically, optimal achievement best may entail the following:

- Personal well-being in a workplace environment, such as the optimal state of resilience, personal resolve, and motivation of a bank employee to overcome difficulties and achieving exceptional KPIs.
- Health functioning on a daily basis, such as an optimal state of health of a senior citizen despite his/her recent temporary illness from the COVID-19 pandemic.

Successful achievement of optimal best in a subject matter, academically or non-academically, is not an easy feat and may, in this analysis, require extensive expenditure of time, effort, cognitive resources, etc. For example, a secondary school student may have to invest in extra financial resources (e.g., to gain additional tutorial support) in order to successfully achieve optimal bests in different academic subjects, which then would enable him/her to enter university and enroll in a desired course. “How much expenditure is enough?” is a personal question that reflects justification, logical decision-making, and sound reasoning (e.g., can a student provide an explanation that could offer a sound justification as to why he/she requires additional financial resources?). Justification, logical decision-making, and sound reasoning are cognitive attributes that may, importantly, associate with the theoretical concept of perceived optimal efficiency.

A desirable feat, of course, would entail and dictate the maximization in an accomplished outcome for the least amount of investment and/or expenditure of time, effort, cognitive resources, etc. An undesirable feat, in contrast, would equate to the minimization in an accomplished outcome for the most amount of investment and/or expenditure of time, effort, cognitive resources, etc. This testament reflects two comparative possibilities: expenditure of time, effort, etc. is “more” than the outcome that would be accomplished vs. the accomplished outcome is “more” than the expenditure of time, effort, etc. We argue that, in this analysis, it is more desirable to have a case where the accomplished outcome is more or greater than the expenditure of time, effort, etc. Our theorization of perceived optimal efficiency (Phan and Ngu, 2021a,b) considers a desirable state of a maximum outcome (i.e., efficiency) and an undesirable state of maximum expenditure (i.e., inefficiency), both of which require and emphasize the importance of personal assessment, judgment, and decision-making. Moreover, of course, we contend that the possibility of a maximum outcome is favored and may, in fact, coincide with a state of comfort. The possibility of maximum expenditure, however, is negative and unfavored, coinciding in this case, perhaps, with a state of discomfort.

### Cognitive Certainty and Perceived Optimal Efficiency

Perceived optimal efficiency, from our point of view, requires systematic planning, organization, and a state of motivation and self-regulation of a person (Zimmerman and Schunk, 2001; Zimmerman, 2002; Wolters, 2003; Boekaerts and Niemivirta, 2005; Schmitz et al., 2007). Systematic planning (e.g., goal setting for the week), organization (e.g., organization of time), and self-regulated behavior (e.g., daily practice of a particular task), for example, may assist a person to minimize his/her expenditure of time, effort, etc. In the context of academic learning, for example, a student may set a number of weekly goals for the next 3 months, use a specific self-regulatory strategy (e.g., the use of monitoring to gauge his/her study patterns), and/or engage in favorable study habits, which could help minimize his/her time, effort, etc. Disorganization, lack of discipline, and unstructured goals, in contrast, are more likely to convolute and/or misdirect a student, resulting in a need for him/her to invest more time, effort, etc.

It would be of interest to consider whether and/or to what extent perceived optimal efficiency could relate to the concept of cognitive entrenchment (Dane, 2010, 2011) and likewise, the proposed conceptualization of cognitive certainty and cognitive uncertainty (e.g., Figure 2). For example, it is plausible that the existing schemas of a person could serve to address and/or compensate for any deficiency in knowledge and understanding, reflecting his/her limited needs to invest in additional time, effort, resources, etc., in order to master the subject content. In other words, from our point of view, fixating on and the use of previous and current knowledge, skills, and understanding may help encourage and facilitate the achievement of optimal efficiency. A change in a course of action for different purposes (e.g., the desire of a person to show creativity), in contrast, may result in a perceived sense of uncertainties (e.g., uncertain of success), which would require a remedy and resolution, involving increased expenditure of time, effort, resources, etc., of a person. Our conceptualization into a state of cognitive certainty and optimal efficiency and, likewise, a state of cognitive uncertainty and inefficiency is shown in Figure 2, where we propose two contrasting pathways: (i) a pathway that depicts the positive impact of expert knowledge and in-depth understanding of a person of his/her personal resolve, decisiveness, and conviction that success or failure would be certain, resulting in a perceived state of comfort, and (ii) a pathway that depicts the negative impact of novice knowledge and limited understanding of a person of his/her ambivalence, indecisiveness, and doubt that success or failure would be certain, resulting in a perceived state of discomfort. This distinction then considers two possible associations:

i. **Efficiency and state of cognitive certainty**. Certainty of success or failure, unlike that of uncertainty, is conclusive and more definitive, reflecting the mental resolute, state of decisiveness, and determination of a person to maintain and/or sustain a well-versed course of action. This testament (e.g., I am certain that I will be successful with this course of action), we contend, suggests that there is an intricate association between a state of cognitive certainty and a perceived state of efficiency. We argue that, in particular, there is the potential “equivalency” and/or association between optimal efficiency and cognitive certainty.
  The equivalency of optimal efficiency and cognitive certainty is interesting as it considers the possibility and the theoretical tenet that an increase in cognitive certainty could also equate to an increase in efficiency, and, by the same token, a decrease in efficiency (i.e., inefficiency) would equate to a decrease in cognitive certainty (i.e., cognitive uncertainty). From our point of view, we acknowledge that there are two possible emphases—namely: (i) self-awareness of the significance of efficiency and/or the insignificance of inefficiency (e.g., that there is a need to be more efficient with time and/or that there are limited resources, which one could use) could serve as an important source of information, guiding, motivating, and/or facilitating a person to be resolute and more decisive in his/her decision-making, and (ii) personal resolute, conviction, and decisiveness in justifying a course of action in terms of success of failure (e.g., I am certain that I will fail if I continue with this course of action), which would give rise to his/her understanding and self-awareness for a need to show efficiency.
ii. Inefficiency and state of cognitive uncertainty. Cognitive uncertainty of success or failure, unlike cognitive certainty, is ambivalent and reflects, importantly, a state of indecisiveness, lack of personal resolve and confidence, and self-doubt of a person about his/her belief to maintain and/or to sustain a course of action. Cognitive uncertainty (e.g., I am uncertain as to whether this change would be successful), we contend, suggests that, perhaps, there is an equivalency and/or an intricate association between a state of cognitive uncertainty and a state of inefficiency—for example, inefficiency is equivalent, or analogous, to a state of cognitive uncertainty.
  The equivalency of inefficiency and a state of cognitive uncertainty, similar to that of the equivalency of optimal efficiency and cognitive certainty, is interesting as it considers two comparative patterns: an increase in cognitive uncertainty would correspond with an increase in inefficiency and likewise, a decrease in inefficiency could equate with a decrease in cognitive uncertainty. This consideration, we contend, may indicate the following understanding between inefficiency and cognitive uncertainty: (i) the indifference of a person to a state of efficiency (i.e., a state of inefficiency) may reflect his/her lack of motivation and state of disorganization, which then could give rise to his/her indecisiveness, lack of confidence and personal resolve, and/or self-determination in decision-making, and (ii) a state of indecisiveness, lack of confidence and personal resolve, and/or strong conviction to be certain (e.g., I am uncertain that this course of action….) could, in effect, negate and/or limit a person from achieving a state of efficiency.

**Figure 3:**
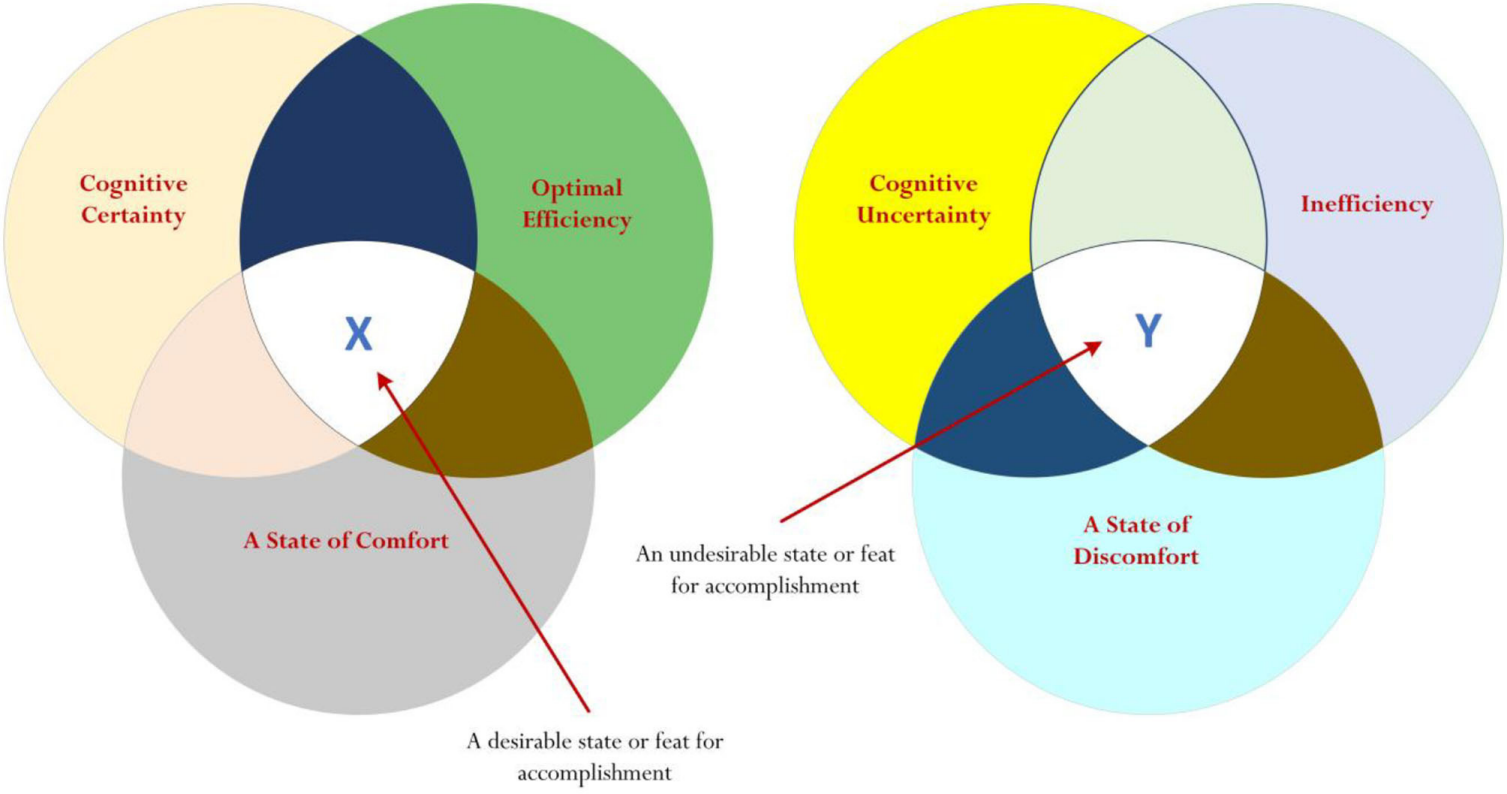
Proposition for consideration: cognitive certainty, optimal efficiency, and state of comfort.

In summary, the preceding sections emphasize a potential relationship between cognitive certainty (and, of course, cognitive uncertainty) and perceived optimal efficiency (and, of course, cognitive uncertainty). Moreover, of course, referring to our earlier discussions (e.g., Figure 2), the equivalency or association between optimal efficiency and cognitive certainty may also intricately coincide with the notion of comfort. This consideration is depicted in Figure 3, which postulates that the nexus between cognitive certainty, optimal efficiency, and a state of comfort (i.e., denoted as “X”) is desirable (e.g., this intersection X is considered as being positive), whereas the nexus between cognitive uncertainty, inefficiency, and a state of discomfort (i.e., denoted as “Y”) is undesirable (e.g., this intersection Y is considered as being negative). Specifically, from Figure 3, and with reference to the concept of cognitive entrenchment (Dane, 2010, 2011), we propose the following:

- Expert knowledge and skills are integral to the accomplishment of a desirable state of X, which is positive, motivational, and proactive. A state of X, which reflects the intersection between cognitive certainty (e.g., the definitive conviction of a person of success), efficiency (e.g., a minimal need to expend human capitals in order to successfully achieve a course of action), and perceived comfort (e.g., a feeling and experience of positivity of a person) may indicate the personal experience and feeling of various *positive life qualities*—such as contentment, ease, and satisfaction. A state of Y, in contrast, is undesirable and would indicate the personal experience and feeling of various *negative life qualities*—for example, discontentment, angst, dissatisfaction, etc.
- Remaining on course without any deviation is encouraged as this cognitive fixation, we contend, would facilitate and strengthen the conviction, personal resolve, and state of decisiveness of a person in his/her decision-making and self-belief that success is definitive. Utilizing existing understanding, knowledge, experiences, etc., likewise, may help advance the progress of a person and/or minimize expenditure of personal resources (e.g., expenditure of time), resulting in a state of efficiency and/or comfort. This theoretical contention, we contend, may explain the case of José Mourinho and his “cognitive fixation” to a specific training methodology, which has brought him immense accomplishments. For example, the personal objective to achieve a state of X (e.g., to achieve and experience contentment), as shown in Figure 3, could and/or would account for the justification and reasoning of José Mourinho to cognitively entrench and to not willingly consider any change in the course of action.

## Conclusion and Implications for Consideration

The study of cognitive entrenchment (Dane, 2010, 2011), theoretically and empirically, is interesting as it provides grounding and personal understanding of the importance of expert schemas. Having expert schemas is beneficial as this would help a person to progress and advance in a course of action. For example, within the context of schooling and academic learning, a secondary school student may utilize his/her in-depth knowledge, understanding, and experience of different pedagogical strategies (i.e., procedural knowledge) (Ngu and Phan, 2016) to help solve complex problems in mathematics. In a similar vein, knowledge in Psychology of a fourth-year undergraduate student may motivate him/her to consider this as a specialization. Having said this, however, there have been discussions, which delve into the potential “negativity” of expertise or expert schemas as opposed to novice schemas. One notable inquiry, in this analysis, relates to the situated fixation of a person to his/her existing schemas or a course of action (Dane, 2010, 2011), which could limit him/her from progressing and advancing. Importantly, aside from advancement in knowledge building and learning experience, situated fixation may also restrict the flexibility, inclination, and/or willingness of a person to adapt to a new context or situation. In terms of academic learning, say, the unwillingness of a university student to deviate and/or change may limit his/her progress in terms of creativity, innovation, exploring new ideas and perspectives, etc. Non-academically, the unwillingness of an architect to consider and/or embrace new building techniques may, likewise, limit his/her creativity in architectural designs.

Our consideration of expert and novice schemas is somewhat different, resulting in our offering of an alternative viewpoint on the theoretical concept of cognitive entrenchment (Dane, 2010, 2011) or the situated fixation of a well-versed course of action of a person. Specifically, as we conceptualized (e.g., Figures 1–3) and argued throughout this article, the unwillingness and/or inflexibility of a person to change, to accept advice to resolve a new context, and/or to explore new frontiers may relate to a number of valid and logical reasons—for example, the person may wish to seek minimize his/her expenditure and/or use of human capitals, or his/her desire to seek a state of comfort, which intricately associates with experiences and feelings of contentment, gratification, etc. Surmising our discussion of a conceptualization is a proposition of a holistic model, as shown in Figure 3, which showcases an important nexus between three major theoretical orientations: cognitive certainty, optimal efficiency, and a state of discomfort. An intersection between cognitive certainty, optimal efficiency, and a state of discomfort, denoted as X in Figure 3, is desirable and, in effect, may justify the reason and purpose of a person for the enactment of cognitive entrenchment.

Our research development into the advancement of the potential positivity of cognitive entrenchment (Dane, 2010, 2011) is not without limitations. Interestingly, for example, in our recent submission of a first draft of this manuscript, one of the reviewers alluded to different extraneous factors, reasons, and/or purposes, which could explain the inclination of a person toward situated fixation of expert schemas. Moreover, from his/her analysis, the reviewer has advised us to consider personal epistemologies, motivational beliefs, other theoretical orientations (e.g., clinical cognitivism), etc. that could, likewise, account for the deliberate intent of a person on a course of action. We appreciate and concur with the insightful comment of the reviewer but acknowledge that our counterargument to the proposition of Dane (2010, 2011), based on philosophical psychology (Thagard, 2014), is still in its early stage of evolution. By all accounts, of course, it is plausible and valid that other theoretical orientations may offer logical and alternative explanations of the case of cognitive entrenchment. Consider, in this analysis, a couple of possibilities: (i) a case whereby a person remains on course without any deviation for reasons and/or purposes other than his/her desire to seek a state of comfort and/or efficiency, (ii) a case whereby a person deviates and changes a course for logical reasons and purposes, which may counter the importance of comfort, efficiency, etc., and (iii) a case whereby a person seeks to capitalize on his/her existing schemas but, at the same time, considers alternative and/or new pathways, which could instill a perception of certainty of success.

### Future Directions for Development

From the preceding sections, it is evident that continuing research development is needed to advance the study of cognitive entrenchment (Dane, 2010, 2011), which, from our point of view, has relevance, potency, and applicability. Changing a course of action, often evident on a daily basis, may pose a conflicting and/or uncomfortable dilemma—for example, will I be successful if I change a course (e.g., a state of cognitive uncertainty)? Or is it better for me to continue on with what I am doing (e.g., a state of cognitive certainty)? There are certain elements that account, motivate, and/or persuade a person to consider one course of action over that of another. Philosophically and drawing from existing research inquiries, we have considered a few elements that we believe could act to influence a person to remain on course without any deviation. What is required, of course, is the appropriate design of a methodological approach that could validate and/or advance our conceptualization for the enactment of cognitive entrenchment—that is, from our point of view, a recommendation for the capitalization and utilization of existing schemas without change. As such, validating the proposition that is detailed in Figure 3, namely the notation of X and/or the notation of Y, would require some form of measurement and assessment of the following:

- The level of willingness, or unwillingness, to change a course of action (e.g., how willing are you that you would your university major?) (i.e., possible indication of the likelihood of a person to engage in cognitive entrenchment).
- The level of mental resolute, confidence, and self-determination of a person in the belief that remaining on course without any deviation for change would yield success (i.e., indication of perceived cognitive certainty of success of a person).
- The willingness of a person vs. his/her reluctance to take risks during the course of learning (i.e., risk-taking is proposed to intricately associate with a state of cognitive certainty).
- Levels of perceived comfort and discomfort (i.e., indication of a feeling of comfort and discomfort).

**Figure 4:**
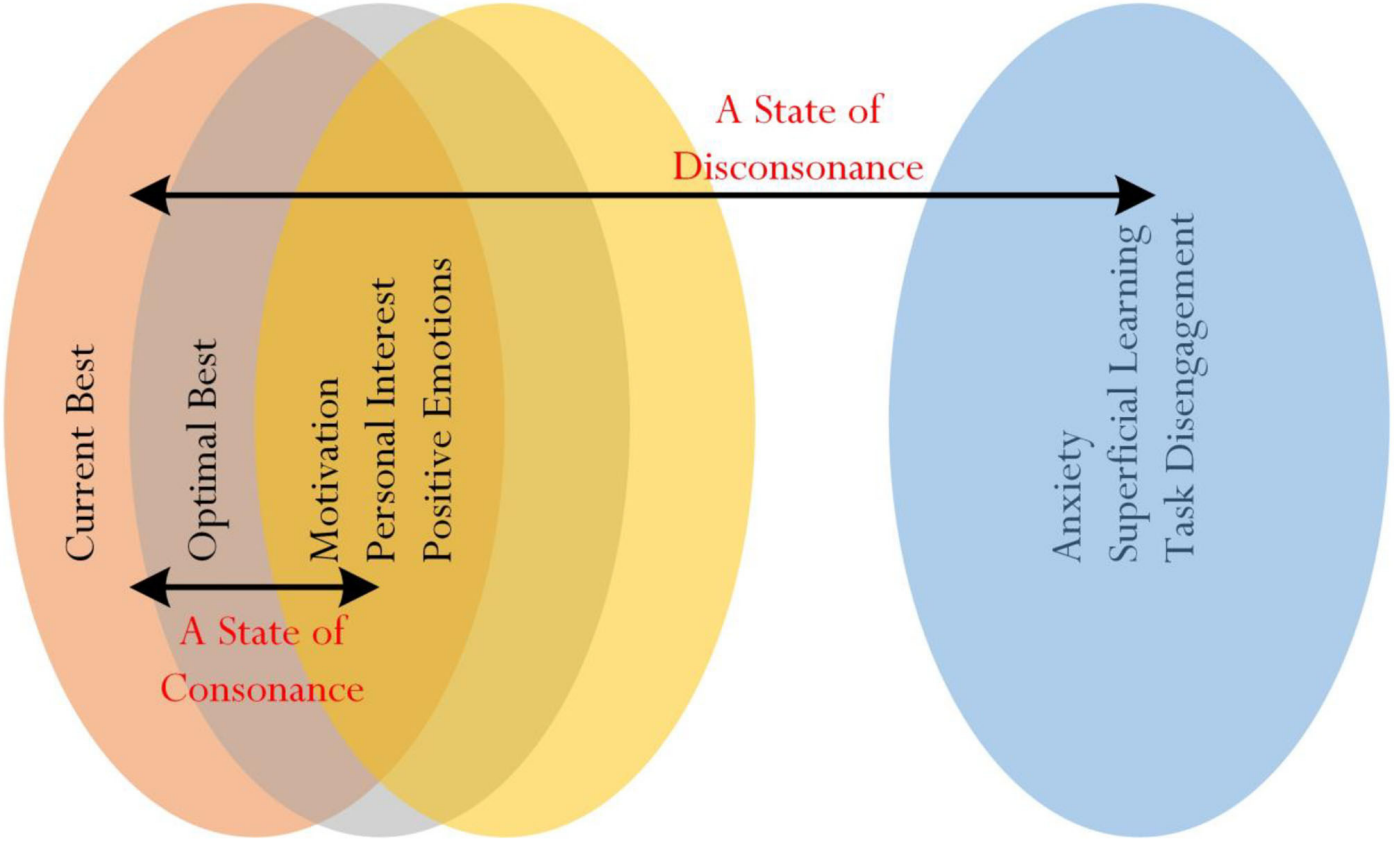
A State of Consonance of Best Practice. Source: Phan and Ngu (2021b).

One possibility from the above consideration is for educators and researchers to use cluster analysis (MacQueen, 1967; Likas et al., 2003; Jain, 2010; Li and Wu, 2012), commonly known as *ClA*, to assist in the identification of “overlapping” of responses between the willingness to deviate, his/her level of mental resolute, confidence, self-determination, and perceived comfort. In our recent non-experimental study that involved Taiwanese university students, for example, we used cluster analysis to explore the nature of optimal best practice (e.g., Phan et al., 2016, 2017, 2019a,b) and more importantly, to propose a theoretical concept, which we termed as a “state of consonance” and a “state of disconsonance” of best practice (Phan and Ngu, 2021b). A state of consonance of best practice, as shown in Figure 4, connotes the potential “clustering” of related variables—for example, optimal best, motivation, and personal interest in learning (i.e., “positive” psychological variables), and anxiety, superficial learning, and task disengagement (i.e., “negative” psychological variables).

**Figure 5:**
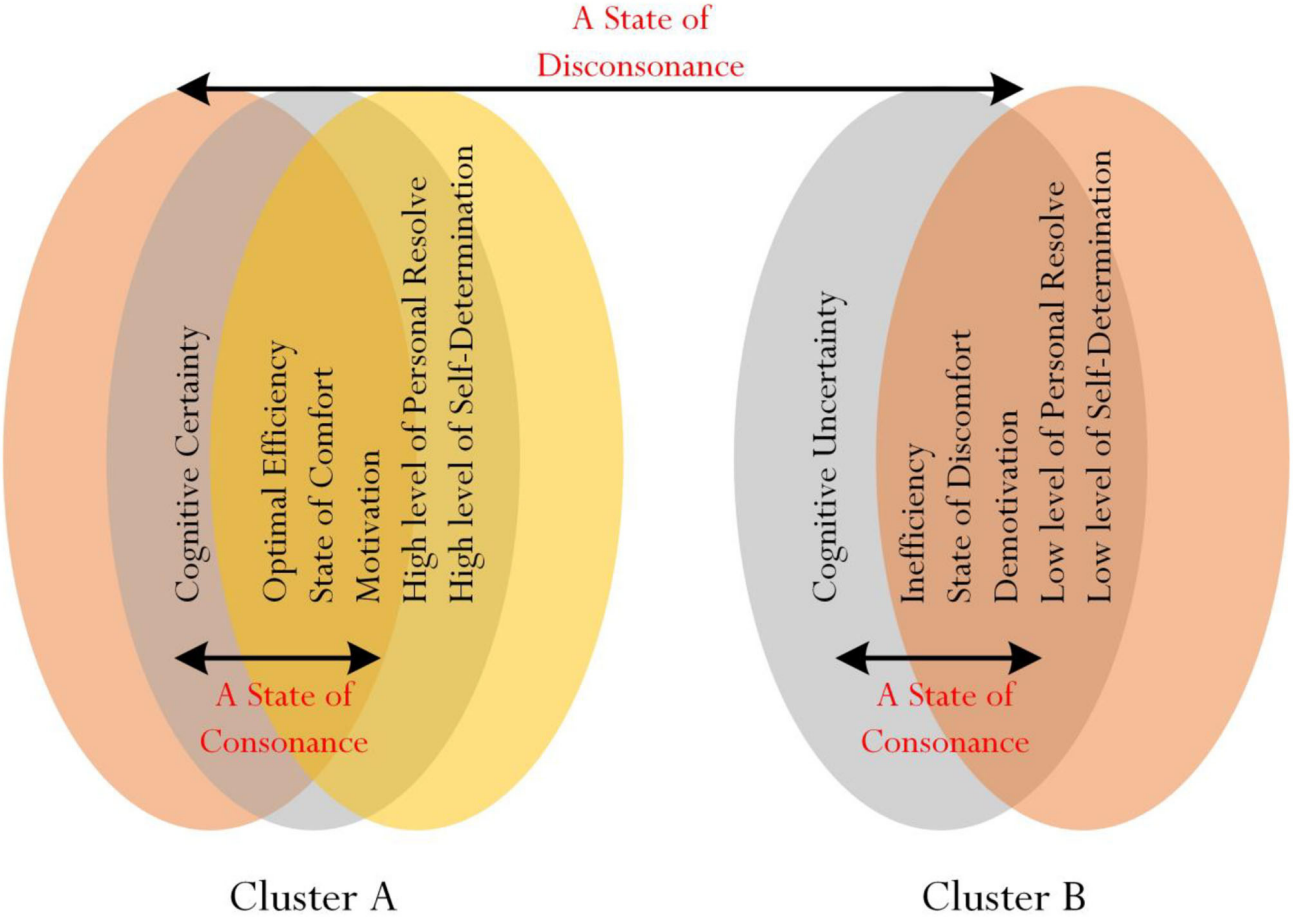
Proposition of clustering for cognitive certainty and cognitive uncertainty.

It is plausible, likewise, to consider a state of consonance of cognitive certainty or cognitive uncertainty and a state of disconsonance between cognitive certainty and cognitive uncertainty (Figure 5). It would be insightful, both in terms of empirical validation and theoretical understanding, for educators and researchers to explore the proposition of statistical clustering as shown in Figure 5. The clustering of university students' responses to Likert-scale measures, for example, may affirm and indicate the following: a state of consonance of cognitive certainty, optimal efficiency, state of comfort, motivation, a high level of personal resolve, and a high level of self-determination (i.e., positive variables) and a state of consonance of cognitive uncertainty, inefficiency, state of discomfort, a low level of personal resolve, and a low level of self-determination (i.e., negative variables). Interestingly, too, we propose a state of disconsonance between the two clusters (e.g., a state of disconsonance between: cognitive certainty and a state of demotivation; cognitive uncertainty and a high level of personal resolve).

An experimental design is unique and quite appropriate for some contexts, especially given that this methodology would allow researchers to gauge causal effects and causal flows (Rogosa, 1979; Marsh and Yeung, 1997; Manolov et al., 2014; Phan and Ngu, 2017). Experimental manipulations, framed as *in situ* interventions in-class, for example, are advantageous and may allow the measurement and assessment of ongoing changes—for example, does the unwillingness, or willingness, of a person to change a course of action (e.g., the continuation of using a particular pedagogical approach of a student) remain steadfast in an in-class intervention (e.g., intervention: a teacher exposes students to a new pedagogical approach)? In a similar vein, could the use of pesuasive feedback encourage personal resolve and conviction of cognitive certainty? It would be of interest then for educators and researchers to consider in-class interventions, which could potentially influence the willingness of a person to change a course of action from T_1_ to T_2_. This “experimental” change, denoted as Δ (willingness to change) _(T1−*T*2)_, we contend, may, indeed, explain the intricacy of cognitive entrenchment. A change in the inclination to change a course of action (e.g., No, I will not change my course of action at T_1_ → Yes, I will change my course of action at T_2_) could, in this case, indicate a state of cognitive entrenchment, whereas, in contrast, the willingness of a person to change, consequently as a result of the persuasive feedback of the teacher would reflect a state of “cognitive dis-entrenchment.”

It is often difficult, for various reasons (e.g., time constraint), to undertake experimental studies in a school or in university. Researchers and educators have consequently resorted to the use of longitudinal, non-experimental designs, which could facilitate and enable the study of growth patterns (Muthén and Curran, 1997; Bollen and Curran, 2006) and temporally displaced predictive effects (e.g., the temporally displaced predictive effect of Variable A at T_1_ on Variable B at T_2_–that is, T_1_ Var A → T_2_ Var B) (Bong, 2001; Harackiewicz et al., 2002; Phan, 2014). Such longitudinal research designs (e.g., the use of multi-wave panel design) could advance theoretical and methodological insights into growth patterns and temporally displaced predictive effects of a state of cognitive certainty or cognitive uncertainty, optimal efficiency or inefficiency, and a perceived feeling of comfort or discomfort (e.g., cognitive certainty at T_1_, which may change to a state of cognitive uncertainty at T_2_, or peceived feeling of discomfort at T_1_, which may change to that comfort at T_2_). It is also valid to consider multiple time points of data collection of the academic performance of a student in a subject matter and his/her corresponding indication of expenditure of time and effort—for example, mathematics quiz (MQ–T_1_) and expenditure of effort (E-T_1_) at T_1_ and mathematics quiz (MQ–T_2_) and expenditure of effort (E–T_2_) at T_2_. A comparison of MQ–T_1_ and MQ–T_2_ [i.e., to measure Δ_(MQ−T1−*MQ*−*T*2)_], referenced in particular against a comparison of E–T_1_ and E–T_2_, may provide fruitful information into the level of expenditure of effort and, hence, perceived state of efficiency of the student. A decrease in Δ_(MQ−T1−*MQ*−*T*2)_ (i.e., indication of underperformance) and an increase in Δ_(E−T1−*E*−*T*2)_ (i.e., indication of increased investment of time and effort) would, in this case, indicate a state of inefficiency.

Finally, as one of our reviewers noted, our attempt to establish a new cognitive framework, which would provide a counterargument to the case of cognitive entrenchment (Dane, 2010, 2011), is not without uncertainty. Some conceptualizations of theoretical orientations (e.g., the theory of human optimization) (Fraillon, 2004; Phan et al., 2017, 2019a), drawn from the use of philosophical psychology (Thagard, 2014), are still “theoretical” as such with limited empirical support. With reference to our aforementioned conceptualization (e.g., Figure 5), for example, it is plausible to consider an alternative argument by which the continuing fixation to a course of action (e.g., the insistence of a university student to continue on with his History Major) does not eventuate into some form of positivity and/or improved results. This contemplation is interesting and, from our point of view, and in tandem with the query of the reviewer, contends a more intricate cognitive structure, which could help explain and account for the complexity of cognitive thoughts and human behaviors in life. In this sense, we acknowledge that our propositions and overall conceptualization for the case of cognitive entrenchment have limitations, requiring further development. As one of our reviewers meticulously mentioned, we cannot be definitive that the effort, personal resolve, confidence, etc., of a person arising from his/her entrenchment to a particular act or course of action would yield successful outcomes.

## Author Contributions

HP and BN contributed equally to the articulation and write-up of this manuscript.

## Conflict of Interest

The authors declare that the research was conducted in the absence of any commercial or financial relationships that could be construed as a potential conflict of interest.

## Publisher's Note

All claims expressed in this article are solely those of the authors and do not necessarily represent those of their affiliated organizations, or those of the publisher, the editors and the reviewers. Any product that may be evaluated in this article, or claim that may be made by its manufacturer, is not guaranteed or endorsed by the publisher.
